# Cardiac H_2_S Generation Is Reduced in Ageing Diabetic Mice

**DOI:** 10.1155/2015/758358

**Published:** 2015-05-11

**Authors:** Sheng Jin, Shi-Xin Pu, Cui-Lan Hou, Fen-Fen Ma, Na Li, Xing-Hui Li, Bo Tan, Bei-Bei Tao, Ming-Jie Wang, Yi-Chun Zhu

**Affiliations:** ^1^Shanghai Key Laboratory of Bioactive Small Molecules and Research Center on Aging and Medicine, Department of Physiology and Pathophysiology, Fudan University Shanghai Medical College, Shanghai 200032, China; ^2^Department of Physiology, Hebei Medical University, Hebei 050017, China; ^3^Department of Pharmacology, School of Pharmacy, Fudan University, Shanghai 201203, China

## Abstract

*Aims*. To examine whether hydrogen sulfide (H_2_S) generation changed in ageing diabetic mouse hearts. *Results*. Compared to mice that were fed tap water only, mice that were fed 30% fructose solution for 15 months exhibited typical characteristics of a severe diabetic phenotype with cardiac hypertrophy, fibrosis, and dysfunction. H_2_S levels in plasma, heart tissues, and urine were significantly reduced in these mice as compared to those in controls. The expression of the H_2_S-generating enzymes, cystathionine *γ*-lyase and 3-mercaptopyruvate sulfurtransferase, was significantly decreased in the hearts of fructose-fed mice, whereas cystathionine-*β*-synthase levels were significantly increased. *Conclusion*. Our results suggest that this ageing diabetic mouse model developed diabetic cardiomyopathy and that H_2_S levels were reduced in the diabetic heart due to alterations in three H_2_S-producing enzymes, which may be involved in the pathogenesis of diabetic cardiomyopathy.

## 1. Introduction

The consumption of soft drinks, which contain high concentrations of fructose, has markedly increased during the last three decades. This has paralleled the increased prevalence of obesity and insulin resistance that are associated with the development of type 2 diabetes and cardiovascular disease [1, 2]. Meta-analyses have also suggested that the consumption of fructose primarily from soft drinks is related to a risk for diabetes [3]. It has been predicted that by 2030, more than 366 million people, among which approximately 196 million people will be between the ages of 60 and 79 years, will be afflicted by type 2 diabetes [4].

The development of diabetic complications, such as cardiovascular disease, is a major cause of mortality among older diabetic patients. As first reported by Rubler et al. [5] in 1972, diabetic cardiomyopathy (DCM) is defined as structural and functional abnormalities in the myocardium in diabetic patients that occurs independently of coronary artery disease and/or hypertension. DCM is characterized by early-onset diastolic dysfunction and late-onset systolic dysfunction. It is a prolonged progression and finally results in heart failure. Multiple mechanisms for the development of DCM have been proposed, including excess oxidative stress, impaired calcium homeostasis, mitochondrial dysfunction, and activation of apoptotic signalling pathways [6–10]. Endothelial dysfunction markedly alters angiogenesis and induces micro- and macrovascular complications in the diabetic heart, which also further contributes to the aetiology of this disease [11]. However, the pathogenesis of DCM remains incompletely understood. Thus, more details are required to further delineate the basic mechanisms underlying DCM. Animal models in DCM research, such as those for drug treatment, high fat diets, and genetic mutations, are commonly used; however, none of the abovementioned models are without limitations. Thus, to better understand the pathogenesis of DCM, a long-term rodent model that possibly mimics human DCM would be advantageous.

Hydrogen sulfide (H_2_S) was recognized as the third gasotransmitter to be identified after nitric oxide and carbon monoxide and is synthesized endogenously from L-cysteine primarily via the action of two enzymes, cystathionine-*γ*-lyase (CSE) and cystathionine-*β*-synthase (CBS) [12–14]. CSE is primarily involved in maintaining cardiovascular function, whereas CBS has an important role in the central and peripheral nervous systems [15, 16]. Recent studies also discovered a third H_2_S producing enzyme, 3-mercaptopyruvate sulfurtransferase (3-MST), which generates H_2_S in the brain as well as in the vascular endothelium [17, 18]. H_2_S has been shown to provide robust protection to various organs after ischemia-reperfusion injury [19–21], stroke [22], and inflammatory disorders [23–25]. In recent years, accumulating evidence derived from cell culture, animal models, and clinical studies suggests that lower H_2_S levels may play a role in the pathogenesis of diabetes mellitus and its associated complications [24, 26, 27]. However, it is not known whether H_2_S generation is changed in ageing diabetic mice with DCM.

Therefore, the aims of this study were to investigate any functional and structural changes in ageing diabetic mouse hearts that resulted from long-term (15 months) feeding of a high-fructose diet and to examine for any changes in the levels of endogenous H_2_S and expression of the three H_2_S-producing enzymes involved in the pathogenesis of DCM in these mice.

## 2. Materials and Methods

### 2.1. Animals and Treatments

Male C57BL/6J mice (8-week-old) from the Department of Laboratory Animal Science, Fudan University were housed at constant temperature (22 ± 2°C) and humidity (60%) with a 12 h dark-light cycle and unrestricted access to food and water. After acclimatization for 2 weeks, mice were randomly divided into two different groups: one group received tap water and the other group received water that contained 30% fructose for 15 months. All our animal experimental procedures were performed according to the Guide for the Care and Use of Laboratory Animals of the National Institutes of Health (NIH) of the United States and approved by the Ethics Committee of Experimental Research, Fudan University Shanghai Medical College.

### 2.2. Glucose Tolerance and Insulin Tolerance Tests

For a glucose tolerance test, glucose levels were measured using glucose strips (Onetouch; Johnson) in blood obtained from the tail vein immediately prior to and at 15, 30, 60, 90, and 120 min after an intraperitoneal (IP) injection of a 25% glucose solution (2 g/kg body weight) into mice that were fasted for 16 h. Insulin sensitivity was tested by IP injection of 0.5 units/kg body weight of recombinant human insulin (Humulin 70/30, Eli Lilly and Company) and plasma glucose measurements were in tail vein blood obtained at 0, 15, 30, 60, 90, and 120 min after this injection in mice that were fasted for 4 h. Areas under the curve (AUC) were determined using the trapezoidal rule.

### 2.3. The 24 h Water and Food Intakes and 24 h Urine Volumes

To record 24 h water and food intakes and collect 24 h urine samples, individual mouse was placed in a metabolic cage (Tecniplast, Italy). Starting 3 days before the collection period, mice were acclimatized to this new environment for 6 h each day.

### 2.4. Echocardiography

To test left ventricular function, mouse two-dimensional echocardiography was performed using a Vevo770 ultrasound device (VisualSonics Inc.), as previously described [28]. Mice were anaesthetized with isoflurane (1%), and M-mode images of the left ventricle were recorded. All measurements were averaged for five consecutive cardiac cycles. Left ventricular internal dimension systole (LVIDs), left ventricular internal dimension diastole (LVIDd), left ventricular end-systolic volume (LVESV), left ventricular end-diastolic volume (LVEDV), ejection fraction and fractional shortening (LVEF and LVFS) were measured to evaluate heart function.

### 2.5. Biochemical Analyses

At the end of the experimental period, mice were fasted for 12 h and then euthanized with 6% chloral hydrate. Blood samples were collected, and their glucose levels were monitored using blood glucose strips (Onetouch; Johnson). Then, plasma was prepared by centrifuging the blood samples at 3000 rpm for 15 min. The plasma levels of triglycerides (TG), total cholesterol (CHO), low density lipoprotein cholesterol (LDL-C), high density lipoprotein cholesterol (HDL-C), blood urea nitrogen (BUN), and creatinine (Cr) were determined by automatic biochemical analyzer (Cobas 6000, Roche). The BUN/Cr index was calculated.

### 2.6. Morphological and Histological Analyses

A heart was surgically removed to determine the heart to body weight ratio (HW/BW × 100%). For histological analysis, the ventricles were excised, fixed in 10% formalin for 48 h before dehydration using a graded ethanol series, embedded in paraffin, sectioned at 4 *μ*m thickness and stained with hematoxylin and eosin (HE) and with Masson's trichrome.

### 2.7. Immunofluorescence Microscopy

Paraffin embedded myocardial tissues were subjected to immunofluorescence for the detection of CBS (Santa Cruz Biotechnology Company) and CD31 (Abcam Company) which were incubated with the antibodies at a dilution of 1 : 100, overnight at 4°C. After washing, the sections were incubated with Alexa Fluor 488 and Alexa Fluor 594 (Life Technologies) secondary antibody at 37°C for 1 h in the dark. Then, sections were incubated with 4′,6-diamidino-2-phenylindole (DAPI) at room temperature for 5 min to stain nuclei. Fluorescent signals were observed under a fluorescence microscope (Olympus).

### 2.8. Western Blot Analysis

Frozen left ventricle tissues were lysed with ice-cold RIPA buffer. Proteins were extracted and quantified using BCA reagent (Shen Neng Bo Cai Corp.). Protein samples were separated on 10% SDS-PAGE gels and transferred to polyvinylidene fluoride (PVDF) membranes (Millipore-Upstate). The membranes were blocked with 5% non-fat milk at room temperature for 1 h and then incubated with antibodies directed against CSE, CBS, 3-MST, Collagen I, Bax, Bcl-2 (Santa Cruz Biotechnology Company), and Collagen III (Abcam Company) at 4°C overnight. After washing with TBST, the membranes were incubated with horseradish peroxidase-conjugated secondary antibodies at room temperature for 1 h. Specific bands were detected with SuperSignal West Pico Chemiluminescent Substrate (Thermo Scientific-Pierce).

### 2.9. Cell Culture and Treatment

Primary neonatal rat cardiac ventricular myocytes (NRCMs) were collected as previously described with some modifications [29]. Briefly, the ventricles of new born Sprague-Dawley rats (1–3 days old) regardless of sex were minced and digested with 0.125% trypsin. Isolated cardiomyocytes were cultured in Dulbecco's modified Eagle's medium/F-12 (DMEM/F12, Life Technologies) supplemented with 10% (v/v) fetal bovine serum (FBS, Thermo Fisher Hyclone), penicillin/streptomycin (100 units), and 0.1 mmol/L 5-bromo-2′-deoxyuridine and maintained in an incubator (37°C with 5% CO_2_). Cells were then cultured in medium containing either normal glucose (5.5 mmol/L, NG group) which served as a normal control or high glucose (33 mmol/L, HG group) for 72 h. Meanwhile, different concentrations (10, 50 and 100 *μ*mol/L) of sodium hydrosulfide (NaHS, a donor of H_2_S) were added in the medium of the HG group, and the control cells were treated with the vehicle. NaHS treatment was repeated every 6 h during the entire treatment period of 72 h. L-Glucose (27.5 mmol/L) was added to medium containing normal glucose (5.5 mmol/L) to make osmotic pressure equal to high glucose.

### 2.10. Cell Viability Assays

The viability of NRCMs which were cultured in 96-well plates was measured by using the Cell Counting Kit-8 (CCK-8) (Dojindo Molecular Technologies), according to the manufacturer's instructions. The absorbance of CCK-8 was obtained with a microplate reader at 450 nm. The values were normalized to the NG group.

### 2.11. Annexin V–FITC/Propidium Iodide Staining for Detecting NRVMs Apoptosis

Cellular apoptosis was determined using the Annexin V–FITC apoptosis detection kit (Dojindo Molecular Technologies), according to the manufacturer's instructions. NRCMs were stained with Annexin V–FITC and propidium iodide (PI) and then the percentage of cell apoptosis was then determined using flow cytometry with a BD FACSCalibur platform (BD Biosciences). The apoptotic ratio was calculated according to the percentage of Annexin V positive apoptotic cells of the total cells. Fluorescent signals were also observed under a laser confocal microscope (Zeiss).

### 2.12. Detection of ROS Levels

ROS levels in NRVMs were determined by dihydroethidium (DHE, Sigma-Aldrich) fluorescence using confocal microscopy. After treatments for 72 h, cells were washed with PBS and incubated with DHE (10 *μ*mol/L) at 37°C for 30 min in the dark. Then, DHE was removed by washing. Fluorescent signals were observed (excitation, 488 nm; emission, 610 nm) under a laser confocal microscope (Zeiss). The values were normalized to the NG group.

### 2.13. Measurement of H_2_S Content

H_2_S levels in plasma, urine, and cell culture medium were measured according to previously described methods [30], and H_2_S levels in heart tissues and NRCMs were measured with some modifications. Briefly, heart tissues and NRCMs were homogenized in ice-cold Tris-HCl (100 mmol/L, pH 8.5) followed by centrifugation at 12,000 g for 20 min at 4°C. Thirty *μ*L supernatant was used to detect H_2_S and proteins in the supernatant were quantified using BCA reagent (Shen Neng Bo Cai Corp.). H_2_S concentrations were determined using a curve generated with sodium sulfide (0–40 *μ*mol/L) standards, and the H_2_S concentrations in plasma, urine, and cell culture medium were expressed as *μ*mol/L. H_2_S concentrations in heart tissues and NRCMs were divided by the protein concentrations and were expressed as *μ*mol/g of protein.

### 2.14. Statistical Analyses

Results were expressed as means ± SEM. Statistical analysis was performed using an SPSS software package, version 13.0 (SPSS, Inc., Chicago, IL, USA). The results for three or more groups were compared using one-way ANOVA followed by Student-Newman-Keuls test. Comparisons between two groups were made using Student's *t*-test. *P* of <0.05 was considered significant.

## 3. Results

### 3.1. Long-Term High-Fructose Feeding Induces Obesity and Type 2 Diabetes in Mice

**Figure 1:**
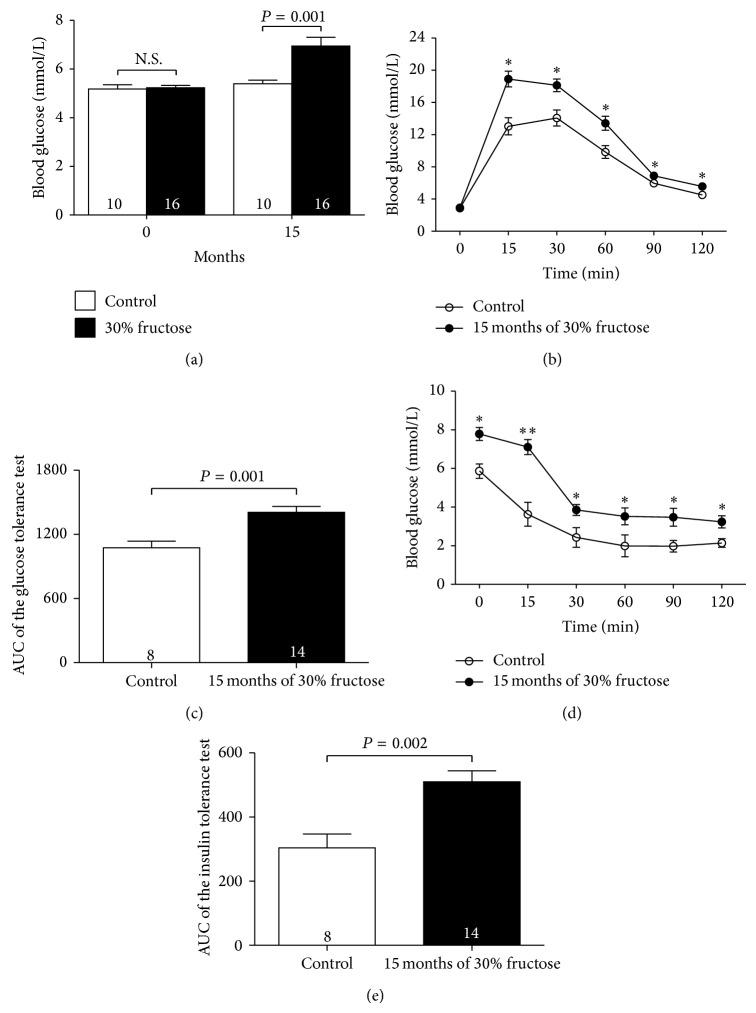
Fifteen months of high-fructose feeding increases fasting blood glucose levels and reduces insulin sensitivity and glucose tolerance in mice. (a) Fasting blood glucose levels of control and high-fructose-fed mice at the beginning and after 15 months of the experimental period. (b) Representative glucose tolerance test curves for control and high-fructose-fed mice after 15 months. (c) Area under the curve (AUC) of glucose tolerance test results was determined for each animal using the trapezoidal rule. (d) Representative insulin tolerance test curves for control and high-fructose-fed mice after 15 months. (e) Area under the curve (AUC) of insulin tolerance test results was determined for each animal using the trapezoidal rule. Results are means ± SEM. ^∗^ *P* < 0.05 versus control; ^∗∗^ *P* < 0.01 versus control.

Compared to mice that were fed water only, mice that were fed a 30% fructose solution for 15 months exhibited characteristics typical of a severe diabetic phenotype, including marked obesity, hyperglycaemia, and dyslipidemia, but there were no differences in BUN and BUN/Cr index (Table 1, Figure 1(a)). The mice that were fed with fructose solution also had increased 24 h urine volumes and water intake at the end of treatment as compared with those of the controls, although there were no differences in their 24 h food intakes (Table 2).

Regarding glucose tolerance and insulin tolerance tests, as expected, mice that were fed the fructose solution developed both impaired glucose tolerance and insulin resistance (Figures 1(b)–1(e)).

### 3.2. Ageing Diabetic Mice Exhibit Cardiac Dysfunction

**Figure 2:**
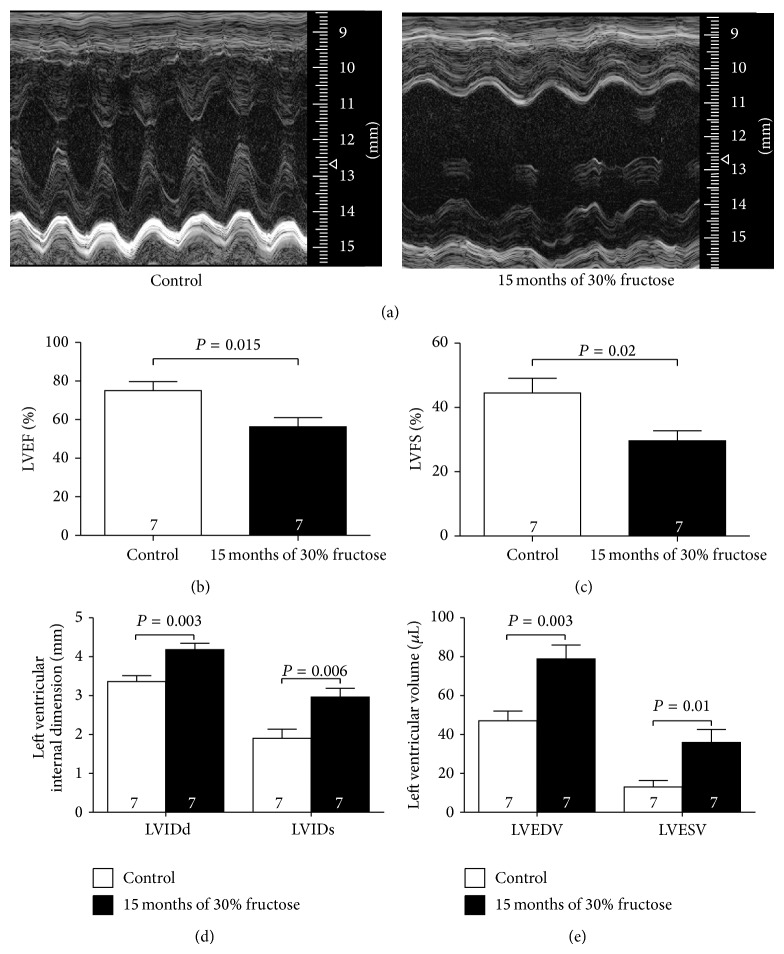
Fifteen months of high-fructose feeding induces cardiac dysfunction. (a) Representative M-mode images. (b–e) Echocardiographic parameter analysis. LVEF, left ventricular ejection fraction; LVFS, left ventricular fractional shortening; LVIDs, left ventricular internal dimension systole; LVIDd, left ventricular internal dimension diastole; LVESV, left ventricular end-systolic volume; LVEDV left ventricular end-diastolic volume. Results are means ± SEM. A *P* of <0.05 was considered significant.

To assess the effects of the long-term high-fructose diet on cardiac function, we used echocardiography to measure cardiac physiological parameters. The representative M-mode images were showed in Figure 2(a). Echocardiography examinations revealed that the left ventricular ejection fraction (LVEF) and fractional shortening (LVFS) were significantly reduced in these mice (Figures 2(b) and 2(c)), whereas the left ventricular internal dimension systole (LVIDs), left ventricular internal dimension diastole (LVIDd), left ventricular end-systolic volume (LVESV), and left ventricular end-diastolic volume (LVEDV) were increased in the high-fructose-induced diabetic mice (Figures 2(d) and 2(e)). These findings indicated that these mice had impaired cardiac function.

### 3.3. Ageing Diabetic Mice Exhibit Cardiac Remodelling and Apoptosis

**Figure 3:**
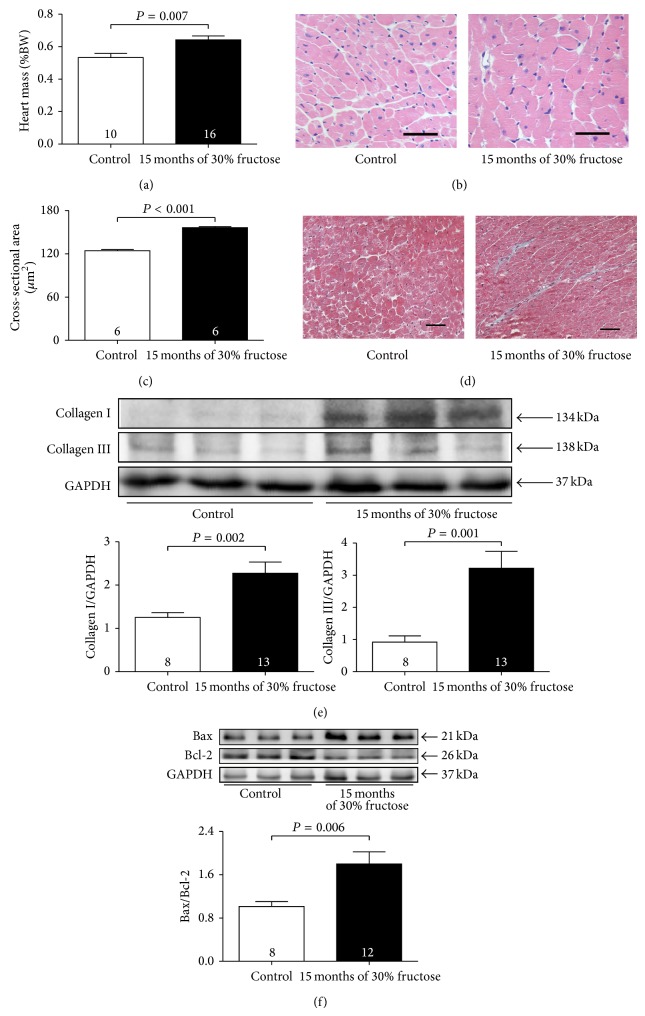
Fifteen months of high-fructose feeding induces cardiac remodelling and apoptosis. (a) Heart to body weight ratio (HW/BW × 100%). (b) Representative HE-stained left ventricular sections (scale bar = 250 *μ*m). (c) Quantitative analysis of cross-sectional areas (CSA). (d) Representative Masson's trichrome-stained left ventricular sections (scale bar = 250 *μ*m). (e) Representative Western blots and quantitative analysis for Collagen I and Collagen III protein expression in the myocardium. GAPDH was used as the internal control. (f) Representative Western blots and quantitative analysis for Bax and Bcl-2 protein expression in the myocardium. GAPDH was used as the internal control. Results are means ± SEM. A *P* of <0.05 was considered significant.

After 15 months of high-fructose feeding, increased HW/BW ratio (Figure 3(a)) and increased cardiomyocyte cross-sectional areas (CSA) were found in the ageing diabetic mice (Figures 3(b) and 3(c)). Masson's trichrome staining showed markedly increased interstitial collagen volumes in these mice as compared with those of the controls (Figure 3(d)). There was also higher expression of Collagen I and Collagen III proteins in these ageing diabetic mice (Figure 3(e)). In addition, Bax/Bcl-2 ratio was also significantly increased in the myocardium of these mice (Figure 3(f)). All of these indicated that myocardial remodelling and apoptosis had occurred in the ageing diabetic mice.

### 3.4. Reduced H_2_S Production in Ageing Diabetic Mice

**Figure 4:**
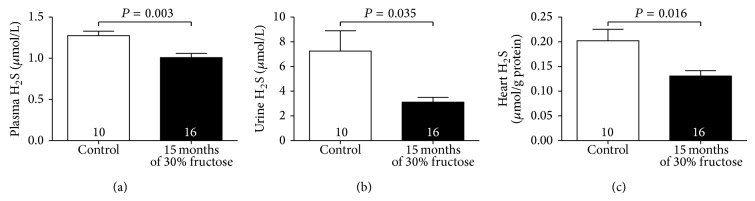
Fifteen months of high-fructose feeding results in reduced H_2_S levels in plasma, urine, and heart tissues. (a) H_2_S levels in plasma, (b) H_2_S levels in urine, and (c) H_2_S levels in heart tissues. Results are means ± SEM. A *P* of <0.05 was considered significant.

In the long-term high-fructose-induced diabetic mice, H_2_S levels in both plasma and urine were significantly lower than those in control mice (Figures 4(a) and 4(b)). We also examined whether H_2_S production was reduced in the diabetic heart. As shown in Figure 4(c), H_2_S production was significantly reduced in the left ventricular tissues of these mice as compared with that in the controls.

### 3.5. Expression of H_2_S-Producing Enzymes Is Altered in Ageing Diabetic Mice

**Figure 5:**
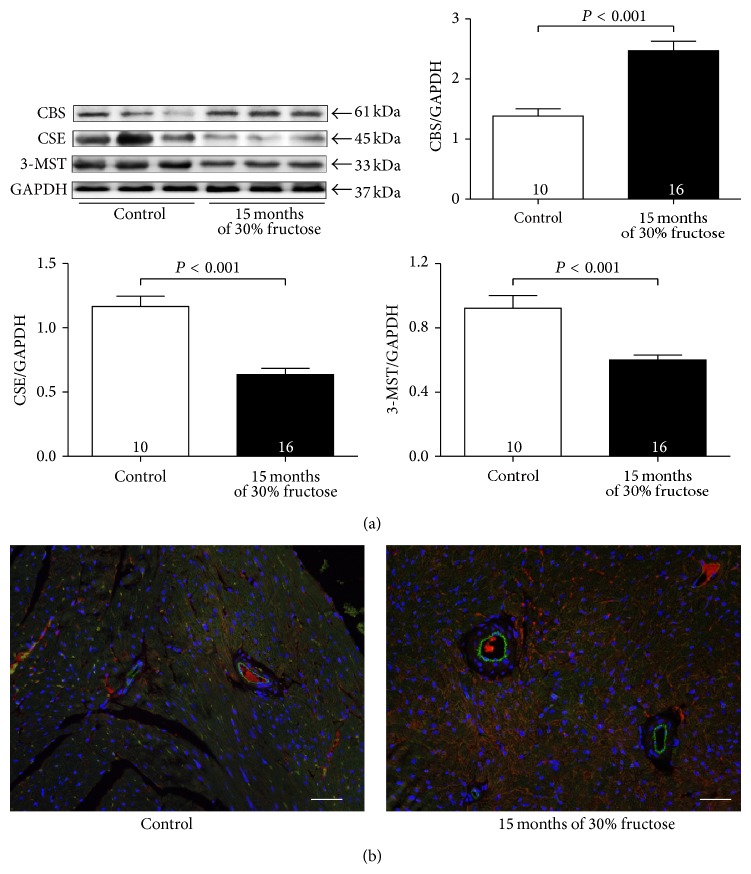
Fifteen months of high-fructose feeding alters CBS, CSE, and 3-MST protein expression. (a) Representative Western blots and quantitative analysis for CBS, CSE, and 3-MST expression in the myocardium. GAPDH was used as the internal control. (b) Representative double-staining immunofluorescence showing the distribution of CBS (red) in the cardiomyocytes and vessels (labelled by CD31, green) from control or ageing diabetic mice (scale bar = 250 *μ*m). Results are means ± SEM. A *P* of <0.05 was considered significant.

Because the H_2_S levels were low in the diabetic heart and H_2_S production depends on CBS, CSE, and 3-MST enzymes, we determined the expression levels of these three enzymes in heart samples. Western blot analysis revealed bands of 61 kDa, 45 kDa, and 33 kDa, which corresponded to CBS, CSE, and 3-MST, respectively. CSE and 3-MST protein expression levels were significantly reduced in the high-fructose-induced diabetic mice as compared with those of controls, whereas CBS protein expression levels were significantly increased in the heart tissues of diabetic mice (Figure 5(a)). The double-staining immunofluorescence showed that CBS protein expression was significantly increased in cardiomyocytes and interstitial but not in coronary vessels (Figure 5(b)).

### 3.6. Reduction of Endogenous H_2_S Involves in High Glucose-Induced Myocardial Injury

**Figure 6:**
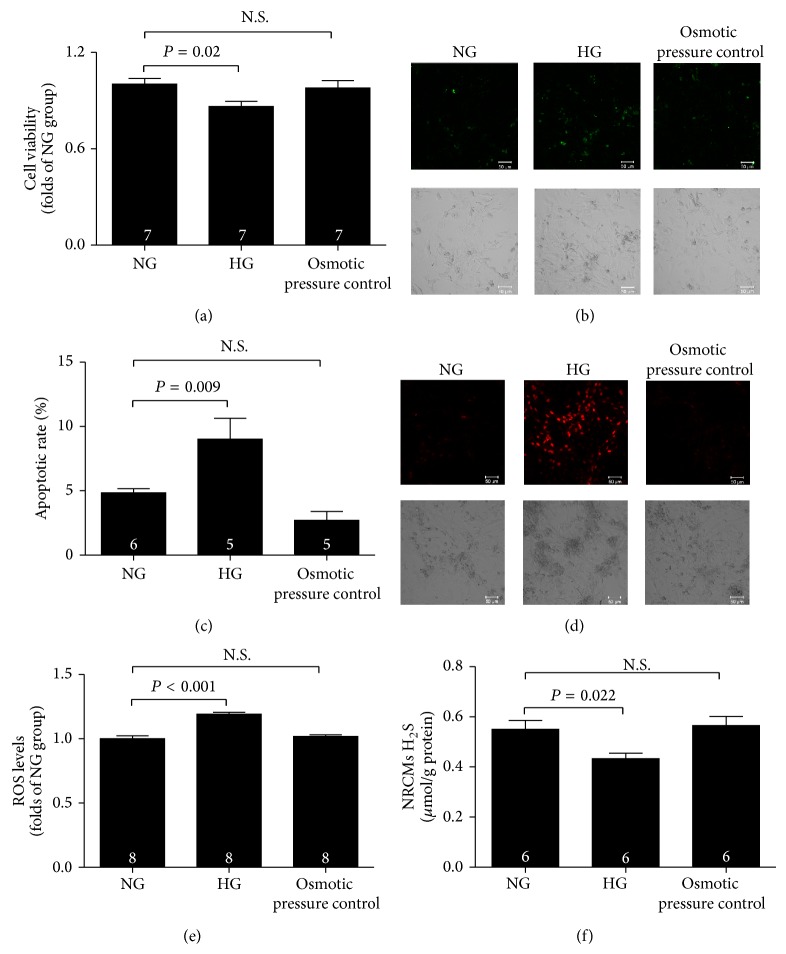
Reduction of endogenous H_2_S involves in high glucose-induced myocardial injury. (a) Neonatal rat cardiac ventricular myocytes (NRCMs) viability measured by CCK-8 assay at the end of the treatment for 72 h. (b) Representative images of cardiomyocyte apoptosis detected by a laser confocal microscope at the end of the treatment for 72 h. (c) Quantitative analysis for cardiomyocyte apoptosis determined by flow cytometry. (d) Representative images of ROS levels in NRCMs detected by a laser confocal microscope at the end of the treatment for 72 h. (e) Quantitative analysis for ROS levels in NRCMs. (f) H_2_S levels in NRCMs at the end of the treatment for 72 h. NG group, normal glucose (5.5 mmol/L); HG group, high glucose (33 mmol/L); Osmotic pressure control group, normal glucose (5.5 mmol/L) + L-glucose (27.5 mmol/L). Results are means ± SEM. A *P* of <0.05 was considered significant.

To confirm whether endogenous H_2_S is involved in the diabetic myocardial injury, NRCMs were incubated in normal glucose (5.5 mmol/L) and high glucose (33 mmol/L) for 72 h to mimic the hyperglycemia in DCM* in vitro*. As shown in Figure 6, NRCMs which were exposed to high glucose resulted in a significantly decreased cell viability (Figure 6(a)), increase in apoptosis rate (Figures 6(b) and 6(c)), and overproduction of ROS (Figures 6(d) and 6(e)). Meanwhile, high glucose-induced myocardial injury was accompanied by a decrease of H_2_S levels in NRCMs (Figure 6(f)).

### 3.7. Exogenous H_2_S Attenuates High Glucose-Induced Myocardial Injury

**Figure 7:**
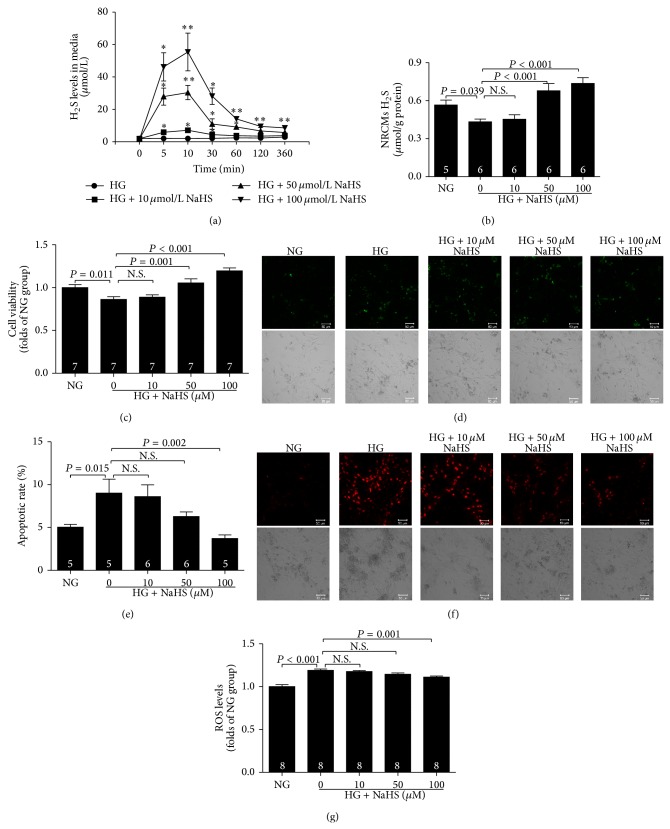
Exogenous H_2_S attenuates high glucose-induced myocardial injury. (a) Exogenous NaHS caused transient increase of H_2_S level in cell culture media within 6 hours. ^∗^ *P* < 0.05 versus HG group; ^∗∗^ *P* < 0.01 versus HG group (*n* = 4 in each group). (b) H_2_S levels in neonatal rat cardiac ventricular myocytes (NRCMs) at the end of the treatment for 72 h. (c) NRCMs viability measured by CCK-8 assay at the end of the treatment for 72 h. (d) Representative images of cardiomyocyte apoptosis detected by a laser confocal microscope at the end of the treatment for 72 h. (e) Quantitative analysis for cardiomyocyte apoptosis determined by flow cytometry. (f) Representative images of ROS levels in NRCMs detected by a laser confocal microscope at the end of the treatment for 72 h. (g) Quantitative analysis for ROS levels in NRCMs. NG group, normal glucose (5.5 mmol/L); HG group, high glucose (33 mmol/L). Results are means ± SEM. A *P* of <0.05 was considered significant.

To determine whether exogenous H_2_S attenuated high glucose-induced myocardial injury, different concentrations (10, 50, and 100 *μ*mol/L) of NaHS (a donor of H_2_S) were added in the media of the HG group, which were repeated every 6 hours during the entire treatment period of 72 h. After adding NaHS in to the cell culture media, H_2_S concentration peaked around 5–30 min and diminished afterwards eventually (Figure 7(a)). H_2_S levels in NRCMs were also increased at the end of treatment (Figure 7(b)). Exogenous H_2_S could suppress the high glucose-induced myocardial injury, leading to an increase in cell viability (Figure 7(c)) and a decrease in apoptotic rate (Figures 7(d) and 7(e)), preventing ROS generation (Figures 7(f) and 7(g)).

## 4. Discussion

In this study, we established an ageing diabetic mouse model by feeding mice water with 30% fructose for up to 15 months to investigate any effects of long-term high-fructose feeding on the mouse cardiovascular system. This resulted in two important findings: (1) long-term high-fructose consumption was associated with diabetic cardiomyopathy (DCM) and (2) H_2_S levels were reduced in the ageing diabetic heart because of alterations in the three H_2_S-producing enzymes.

Despite recent advances in care and management, diabetes and its associated complications continue to be a major global public health problem, which is gradually worsening, particularly in the developing nations. Although genetic predisposition is an important aetiology of this disease, environmental factors, such as diet and physical activity, are also involved. In particular, long-term consumption of overly nutritious diets that are enriched in fructose and fats can cause initiation of obesity and insulin resistance, which result in development of type 2 diabetes and its associated complications [31, 32].

Although increased coronary atherosclerosis is the major cause of death among diabetic patients, particularly elderly patients, there is an increased risk for the development of heart failure that is independent of coronary artery disease and hypertension. This adverse situation is referred to DCM, which is characterized by cardiac remodelling, fibrosis, progressive cardiac dysfunction and independent of coronary artery disease [33, 34]. However, therapeutic strategies to effectively prevent or reduce diabetic heart failure are still unavailable because of our incomplete understanding of the underlying mechanisms. Thus, an animal model that can mimic the extremely protracted pathogenesis of DCM is required.

Animal models in DCM research are quite common. However, the drawback of these models is that they only mimic a short term for DCM but do not mimic it long term. Thus, to better understand the pathogenesis of DCM, a long-term rodent model mimicking as best as possible human DCM would be of great help. In this study, ageing diabetic mice were induced by feeding with a 30% fructose water solution for 15 months (at the end, mice were 17 months old), and these mice were overweight, hyperglycaemic, insulin resistant, and dyslipidemic by the end of the study.

This long-term fructose feeding also caused morphological changes in mouse heart tissue, increased interstitial collagen deposition and expression and increased heart/body weight ratios, indicative of cardiac hypertrophy and fibrosis. The increased Bax/Bcl-2 ratio also indicated cardiomyocyte apoptosis in these mice. M-mode echocardiography confirmed that LVEF and LVFS were significantly reduced along with an increased LV volume, which suggested hyperglycaemia-induced cardiac dilation and dysfunction. In contrast, mice that were fed tap water only for the same period remained healthy. This was consistent with the results of previous reports that showed the developmental stages of cardiomyopathy in db/db diabetic mice [35, 36].

A number of mechanisms have been proposed to contribute to the development of diabetic cardiomyopathy, including increased oxidative stress [37], altered calcium homeostasis [38], activation of apoptotic signals [39], and reduced angiogenesis [40]. H_2_S is the third gasotransmitter to be identified after nitric oxide and carbon monoxide, and it is endogenously generated by three enzymes: CBS, CSE, and 3-MST. H_2_S is involved in numerous pathophysiological and physiological functions due to its antiapoptotic [20], antioxidative [21] anti-inflammatory [41, 42], and proangiogenic activities [43, 44] in mammals, and reduced endogenous H_2_S levels are related to various diseases. However, information on endogenous H_2_S levels in the hearts of ageing diabetic mice with DCM is fairly limited. Therefore, it would be premature to conclude whether the change in H_2_S generation in the ageing diabetic mouse heart is involved in DCM. Our results indicated that circulating, heart and urine H_2_S levels in long-term high-fructose-fed diabetic mice were lower than those in control mice, which was similar to the results of previous studies of diabetic patients and animals [24, 45, 46]. To confirm whether endogenous H_2_S is involved in the diabetic myocardial injury, NRCMs were exposed to high glucose (33 mmol/L) for 72 h to mimic the hyperglycemia in DCM* in vitro* and resulted in a significant decrease in cell viability, increase in apoptosis rate, and overproduction of ROS, accompanied by a decrease of H_2_S levels. Exogenous H_2_S could suppress the high glucose-induced myocardial injury by preventing ROS generation, inhibiting cardiomyocyte apoptosis and promoting cell viability.

CSE, a key enzyme involved in H_2_S production in the cardiovascular system, was downregulated, which might have contributed to the reduced H_2_S levels. These findings were consistent with those in previous studies. Zhang et al. reported that glucose induced SP1 phosphorylation via p38 MAPK activation, which resulted in decreased CSE promoter activity and the subsequent downregulation of the expression of CSE gene [47]. Notably, for the first time, we report that 3-MST protein expression was also reduced in long-term high-fructose-fed diabetic mice. CBS was reported to be mainly expressed in nervous system, but this enzyme has also been shown to exist in the cardiovascular system [48]. In our study, CBS existed in the cardiac tissue and was upregulated after the long-term fructose feeding. The double-staining immunofluorescence further showed that CBS protein expression was significantly increased in cardiomyocytes and interstitial but not in coronary vessels. Although CBS was also reported to be upregulated in some studies [42, 49], we considered that CBS upregulation might be due to a compensatory response for hyperhomocystinaemia (HHcy). High-fructose-fed diabetic mice typically also have HHcy [50]. In the trans-sulfuration pathway, homocysteine (Hcy) condenses with serine to form cystathionine, which is catalyzed by CBS. To metabolize this excess Hcy, CBS is upregulated; meanwhile, Hcy level can be lowered by inducing transgenic human CBS (Tg-hCBS) [51]. However, this increase in CBS is not sufficient to cause an increase in overall H_2_S generation, because the expression of both CSE and 3-MST, the other two H_2_S-producing enzymes in cardiovascular system, are downregulated, and the defined mechanisms underlying CBS upregulation remain to be further studied.

As discussed above, there are conflicting reports regarding the regulation of H_2_S-producing enzymes in diabetes. Several* in vivo* studies have also reported different protein expression levels for CSE or CBS in various tissues. There were reductions in both CSE protein expression and CSE activity, which could have resulted in impaired H_2_S levels both in the liver tissues of STZ-treated T1D rats and PBMCs isolated from T1D patients [52]. CBS and CSE were also lower in hyperglycaemic Akita mice [53]. Yamamoto et al. reported that CSE expression was markedly reduced in the diabetic kidney, whereas CBS expression was unaffected in the proximal tubules of diabetic kidneys in CaMTg mice [54]. However, Suzuki et al. did not find any notable changes in the expression of CSE or CBS in the brain, heart, kidney, lung, liver, or thoracic aorta of rats subjected to STZ-induced diabetes [55]. On the other hand, several studies suggested that the expression of CBS and/or CSE was increased in the pancreas, liver, and kidney of STZ-diabetic rats [56, 57]. Similar conflicting reports regarding the protein expression of H_2_S producing enzymes based on* in vitro* studies can be found [58–60].

These conflicting findings on the expression of H_2_S-producing enzymes may be due to the different responses of different organs and different cell types. This may also depend on the stage or severity of the disease. These questions as well as the actual molecular regulation of these enzymes need to be further investigated. Despite the controversy on the expression of the three H_2_S-producing enzymes, it appears that endogenous H_2_S plays an important role in the development of diabetes and its complications.

## 5. Conclusion

In conclusion, our results suggest that ageing diabetic mice induced by long-term high-fructose consumption developed diabetic cardiomyopathy and that H_2_S levels were reduced in the diabetic heart due to alterations in the expression of the three H_2_S-producing enzymes, which might be involved in the pathogenesis of DCM.

## Figures and Tables

**Table 1:** Physiological and biochemical results for control and ageing diabetic mice induced by a 30% fructose solution fed for 15 months.

	Control (*n* = 10)	30% Fructose (*n* = 16)
Body weights (g)	27.90 ± 0.72	34.19 ± 0.70^**^
Total cholesterol (mM)	1.54 ± 0.04	2.00 ± 0.06^**^
Triglycerides (mM)	0.46 ± 0.04	0.38 ± 0.02
HDL cholesterol (mM)	1.13 ± 0.06	1.30 ± 0.07
LDL cholesterol (mM)	0.26 ± 0.02	0.47 ± 0.04^**^
BUN (mM)	6.68 ± 0.49	6.07 ± 0.34
Cr (*μ*M)	9.56 ± 0.5	7.54 ± 0.42^**^
BUN/Cr index	0.7 ± 0.04	0.82 ± 0.05

Results are means ± SEM. ^**^ *P* < 0.01 versus control; HDL, high density lipoprotein; LDL: low-density lipoprotein; BUN: blood urea nitrogen; Cr: creatinine; BUN/Cr blood urea nitrogen/creatinine.

**Table 2:** Twenty-four-hour metabolic characteristics of control and ageing diabetic mice induced by a 30% fructose solution fed for 15 months.

	Control (*n* = 9)	30% Fructose (*n* = 16)
24 h water intake (mL)	3.74 ± 0.31	6.04 ± 0.38^**^
24 h food intake (g)	0.62 ± 0.19	0.32 ± 0.15
24 h urine volume (mL)	1.02 ± 0.16	2.13 ± 0.25^**^

Results are means ± SEM. ^**^ *P* < 0.01 versus control.
